# Dereplication and Quantification of Major Compounds of *Convolvulus arvensis* L. Extracts and Assessment of Their Effect on LPS-Activated J774 Macrophages

**DOI:** 10.3390/molecules27030963

**Published:** 2022-01-31

**Authors:** Hafiz Abdul Khaliq, Sergio Ortiz, Mireille Alhouayek, Giulio G. Muccioli, Joëlle Quetin-Leclercq

**Affiliations:** 1 Pharmacognosy Research Group, Louvain Drug Research Institute, UCLouvain, 1200 Brussels, Belgium; hafiz.abdulkhaliq@uclouvain.be (H.A.K.); sergio.ortiz@uclouvain.be (S.O.); 2 Bioanalysis and Pharmacology of Bioactive Lipids Research Group, Louvain Drug Research Institute, UCLouvain, 1200 Brussels, Belgium; mireille.alhouayek@uclouvain.be (M.A.); giulio.muccioli@uclouvain.be (G.G.M.); 3 Department of Pharmacognosy, Faculty of Pharmacy, Bahauddin Zakariya University, Multan 60800, Pakistan

**Keywords:** field bindweed, GNPS, molecular networking, HPLC-UV-HRMS, phenolics, inflammation, biological screening

## Abstract

*Convolvulus arvensis* is used in Pakistani traditional medicine to treat inflammation-related disorders. Its anti-inflammatory potential was evaluated on hexane, dichloromethane, ethyl acetate, methanol, and aqueous extracts of whole plant on pro-inflammatory mediators in LPS-activated murine macrophage J774 cells at the non-cytotoxic concentration of 50 µg/mL. Ethyl acetate (ARE) and methanol (ARM) extracts significantly decreased mRNA levels of IL-6, TNF-α, MCP-1, COX-2, and iNOS. Furthermore, both extracts dose dependently decreased IL-6, TNF-α, and MCP-1 secretion. Forty-five compounds were putatively identified in ARE and ARM by dereplication (using HPLC-UV-HRMS^n^ analysis and molecular networking), most of them are reported for the first time in *C. arvensis*, as for example, nineteen phenolic derivatives. Rutin, kaempferol-3-*O*-rutinoside, chlorogenic acid, 3,5-di-*O*-caffeoylquinic acid, *N*-*trans*-*p*-coumaroyl-tyramine, and *N*-*trans*-feruloyl-tyramine were main constituents identified and quantified by HPLC-PDA in ARE and ARM. Furthermore, chlorogenic acid, tyramine derivatives, and the mixture of the six identified major compounds significantly decreased IL-6 secretion by LPS-activated J774 cells. The activity of *N*-*trans*-*p*-coumaroyl-tyramine is shown here for the first time. Our results indicate that ARE, ARM and major constituents significantly inhibited the expression of pro-inflammatory mediators, which supports the use of this plant to treat inflammatory diseases.

## 1. Introduction

*Convolvulus arvensis* L. belonging to the family Convolvulaceae is among the very frequently used plants in the traditional medicine of Pakistan to treat inflammatory conditions [1]. *C. arvensis* is a perennial deep-rooted creeping weed found in temperate regions throughout the world. Its common English name is “field bindweed”, while in Pakistan it is locally called “leli” or “wanveri” [2,3].

In the traditional medicine of Pakistan, roots of *C. arvensis* are used as purgatives [4], while the leaves’ paste is applied topically to treat boils, inflammation, and rheumatism [3,5,6]. To cure constipation, dried whole plant mixed with molasses is given with milk at night or fresh whole plant boiled in water is eaten as a vegetable with wheat bread [6,7]. Fresh plant ground with black peppers in water is given to treat bleeding piles, leprosy, and other skin diseases [3,6]. *C. arvensis* is reported to possess antioxidant [8], antiarthritic [9,10], hepatoprotective [11], and hypoglycemic [12] activities. Phytochemical investigation has shown the presence of tropane alkaloids [13], coumarins [12], resin glycosides [14], flavonoids [15], phenolic acids [8], and phytosterols [16] in *C. arvensis*.

Inflammation is a body’s natural defense system triggered by a variety of harmful stimuli such as damaged cells, pathogens, lipopolysaccharides, or irritants. Immune cells, especially macrophages, release biochemical mediators, such as pro-inflammatory cytokines (e.g., IL-6 and TNF-α) and chemokines (e.g., MCP-1), to coordinate the immune reaction that will eliminate the inflammatory triggers and promote tissue repair and recovery [17]. Likewise, cyclooxygenase 2 (COX-2) and inducible nitric oxide synthase (iNOS) are two important enzymes involved in the inflammatory process [18,19]. However, uncontrolled inflammation may lead to severe disorders such as inflammatory bowel diseases, asthma, rheumatoid arthritis, and neurodegenerative diseases [20].

Traditional medicines are a rich source of new drugs [21]. The phytochemicals that could be responsible for the biological activity of plants in traditional medicine need to be identified and quantified, notably to prepare standardized crude extracts or to isolate them to develop into a new drug. To avoid the isolation of already known compounds, “dereplication” is now extensity used. MS along with the Global Natural Product Social Molecular Networking (GNPS) (http://gnps.ucsd.edu) is getting popular to dereplicate known compounds of natural products [22].

Due to the very frequent use of *C. arvensis* in Pakistani traditional medicine to treat inflammatory conditions, we evaluated this plant and we report the inhibitory effect of *C. arvensis*’ extracts on the expression of pro-inflammatory markers in LPS-stimulated J774 macrophage. Furthermore, we describe the dereplication of the major most active crude extracts chemical constituents performed by HPLC-MS/MS and molecular networking. Finally, we report the quantification of their major constituents by HPLC-PDA.

## 2. Results and Discussion

### 2.1. Extraction Yield and MTT Assay of Crude Extracts

We prepared four crude extracts from *C. arvensis* by successive Soxhlet extraction with four solvents of increasing polarity, namely hexane, dichloromethane, ethyl acetate, and methanol (ARH, ARE, ARD, and ARM, respectively), and a decoction (ARW). The purpose of using these solvents was to extract as much chemical constituents as possible and to divide the chemical constituents in four parts depending on their solubility: non-polar to polar, from hexane to methanol. In addition, a decoction was prepared to dissolve the water-soluble constituents and also because, in general, traditional use of plant-based therapies involves their preparation in water, mostly as decoctions. Extraction yields of ARH, ARD, ARE, ARM, and ARW were 1.84%, 2.28%, 0.66%, 9.86%, and 14.31%, respectively. The highest yield of ARW indicates the presence of a high quantity of polar compounds in *C. arvensis*, probably primary metabolites, which are soluble in water. Yields of ARH, ARD, and ARE are very low, compared to ARM, indicating the lower presence of non-polar (fatty) and less polar (e.g., terpenoids) substances. Methanol is a very good solvent for extraction because it can extract both hydrophilic, but also moderately lipophilic substances that are present in higher quantities in this plant. This could explain the relatively high yield of ARM.

**Figure 1:**
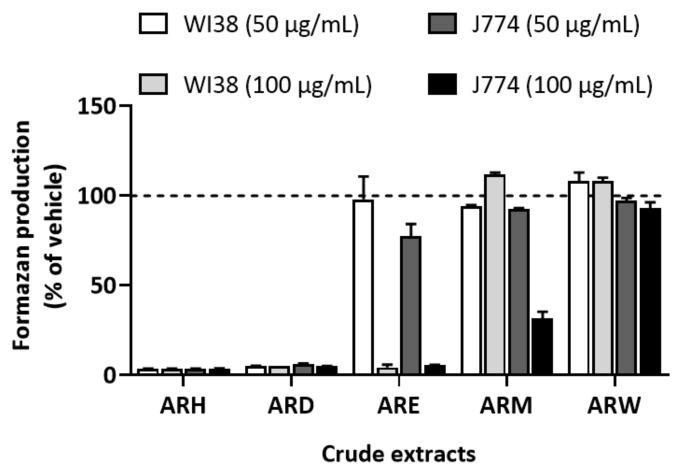
**Effect of the crude extracts on cell viability.** WI38 and J774 cells were treated with 50 and 100 µg/mL of the crude extracts or vehicle (DMSO and EtOH-H_2_O (25:75)) for 72 h. Then, an MTT assay was performed. Data are expressed as mean ± SEM. *n* = 2 in triplicates.

The effect of these five extracts on cellular metabolic activity, as a proxy of cytotoxicity, was measured using an MTT assay on two cell lines, namely WI38 and J774. As cytotoxicity varies according to the type of cells, we decided to test two cell lines: J774, the line used for the tests on cytokines production and WI38, to verify that the eventual effects observed on J774 were not specific to this cell line. As frequently performed with crude extracts, two concentrations of these extracts were used, 100 µg/mL and 50 µg/mL. Camptothecin was used as positive control. The IC_50_ values obtained for camptothecin were 34.2 ± 4.9 ng/mL and 7.1 ± 0.6 ng/mL for WI38 and J774 cells, respectively. When tested at 100 µg/mL, most of the extracts decreased the MTT reduction into formazan in both cell lines (Figure 1). At 50 µg/mL concentration, ARH, ARD, ARE, ARM, and ARW showed percentage viability of 3.6 ± 0.2, 5.1 ± 0.1, 98.1 ± 12.7, 94.1 ± 0.8, and 108.4 ± 4.7 for WI38 cells, and 3.7 ± 0.1, 6.0 ± 0.5, 77.5 ± 6.8, 92.7 ± 0.4, and 97.3 ± 1.6 for J774 cells, respectively. The difference in cytotoxicity of the different crude extracts is explained by the presence of different types of compounds in these extracts. ARE, ARM, and ARW, non-cytotoxic at the tested concentrations, were selected to evaluate their effect on LPS-stimulated J744 cells.

### 2.2. Effect of ARE, ARM, and ARW on the Expression of Pro-Inflammatory Mediators in LPS-Stimulated J744 Cells

**Figure 2:**
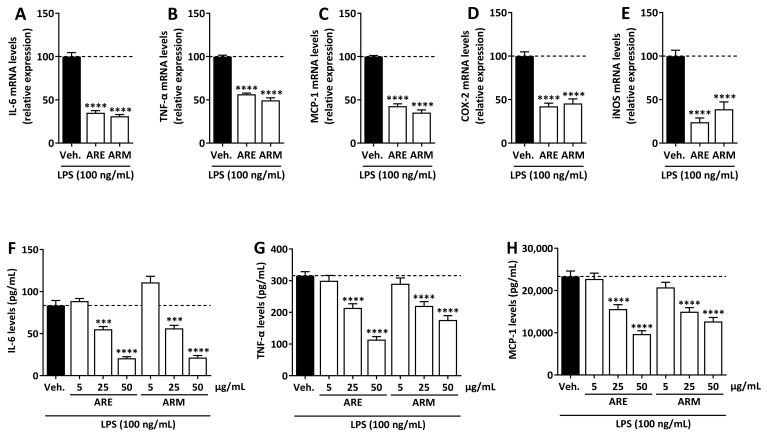
**Effect of ARE and ARM on the expression of pro-inflammatory mediators in LPS-stimulated J744 cells.** (**A**–**E**) Cells were incubated with crude extracts (50 µg/mL), or vehicle (Veh., 0.25% DMSO) and LPS (100 ng/mL) for 8 h. mRNA levels of IL-6, TNF-α, MCP-1, COX-2, and iNOS were analyzed by RT-qPCR with RPL19 used as reference gene. Results are expressed in percentage of the LPS-vehicle condition. (**F**–**H**) Cells were incubated with crude extracts, or vehicle (Veh., 0.25% DMSO) and LPS (100 ng/mL) for 8 h. IL-6, TNF-α, and MCP-1 protein levels in the supernatant medium were quantified by ELISA. IL-6, TNF-α, and MCP-1 were not detected in the medium of the unstimulated, vehicle-treated cells. The data were analyzed by the one-way ANOVA followed by Dunnet’s post hoc test for comparisons between groups, are expressed as mean ± SEM. *n* = 3 in triplicates, *** *p* < 0.001, **** *p* < 0.0001 vs. Veh.

The effect of ARE, ARM, and ARW was analyzed on LPS-induced J774 cell activation by assessing the expression of cytokines (IL-6, TNFα, and MCP-1), COX-2, and iNOS following the incubation of cells with the crude extracts (50 µg/mL) and LPS (100 ng/mL) for 8 h. IL-6 and TNF-α are not only key pro-inflammatory cytokines of innate immune response, but can also amplify the inflammation to a chronic state. IL-6 plays a key role in regulating Th17 cells (Th17) and regulatory T cells (Treg) response, the former cell type leading to autoimmune disorders while the latter cell type counters its effects [23]. TNF-α augments the transcription of other pro-inflammatory cytokines [24], while MCP1, also referred to as CC chemokine ligand 2 (CCL2), is a chemokine favoring monocytes recruitment [25]. COX-2 is an enzyme that mediates the synthesis of prostaglandins, which are considered as important mediators of the innate immune response [26]. iNOS causes increased production of NO, leading to oxidative stress, tissue damage, and inflammation [19]. First mRNA levels of IL-6 were analyzed by RT-qPCR. ARW did not decrease mRNA levels of IL-6 (data not shown here), while ARE and ARM significantly inhibited the LPS-induced expression of IL-6 mRNA (Figure 2A). The effect of ARE and ARM was further analyzed on TNF-α, MCP-1, COX-2, and iNOS mRNA expression. ARE and ARM significantly inhibited TNF-α, MCP-1, COX-2, and iNOS expression (Figure 2B–E). To confirm at the protein level the effects of ARE and ARM, IL-6, TNF-α, and MCP-1 levels in the culture medium were measured as well. Both ARE and ARM dose-dependently decreased LPS-induced IL-6, TNF-α, and MCP-1 secretion by J774 cells, further supporting their interesting properties (Figure 2F–H).

### 2.3. Dereplication of ARE and ARM

**Figure 3:**
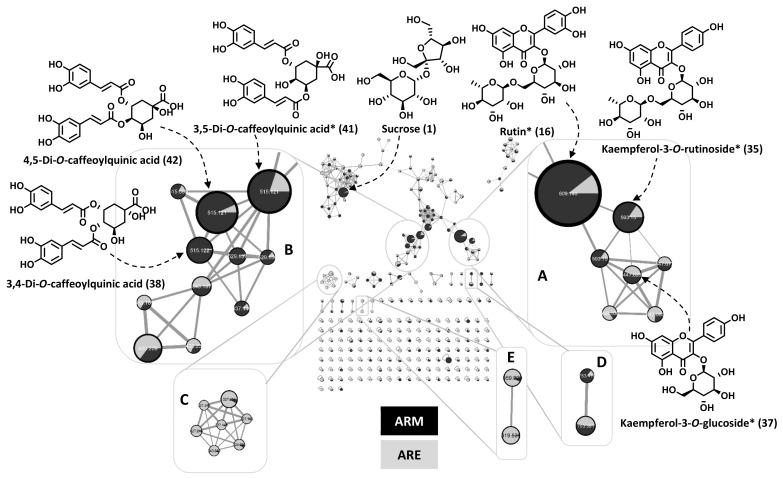
**Molecular network of *C. arvensis* extracts (ARE and ARM).** A: flavonoid glycosides; B: phenolic acids; C: lipid acids, D: flavonoid triglycosides; E: lipid glycosides. Clusters were built with a cosine of 0.7 with a minimum of 4 matched peaks. Size of nodes are proportional to corresponding peak area. Edge width are proportional to the corresponding cosine value. Compounds marked with * were additionally identified by standard comparison.

In order to start exploring the compounds that could potentially mediate the observed effects, we aimed at dereplicating ARE and ARM extracts by HPLC-MS/MS along with the Global Natural Product Social Molecular Networking (GNPS). Previous phytochemical studies have shown that *C. arvensis* contains alkaloids, phenolic acids, flavonoids, sterols, resin glycosides, coumarins, and triterpenes [27]. Based on our LC-UV-MS^n^ chromatographic analysis of ARE and ARM, mainly developed to analyze phenolic compounds, which are known to be good anti-inflammatory candidates [28], the main detected metabolites can be organized in three metabolites groups: phenolic acids, flavonoid glycosides, and glycolipids (Table 1). The molecular network of both extracts organizes the fragmented compounds in several clusters, corresponding to phenolics/glycosidic compounds (Figure 3A,B), lipid acids (Figure 3C), triglycosides flavonoids (Figure 3D), and glycoside lipids (Figure 3E). After several sugars, derivatives and small organic molecules, the first eluted phenolic acid was putatively identified as *O*-glucosyl-caffeic acid (**10**), which gave pseudo-molecular ion [M − H]^−^ at *m*/*z* 341 and fragmented to the corresponding aglycon, which gave a signal at *m*/*z* 179. The same fragment was observed for the phenolic acids identified as chlorogenic acid (3-*O*-caffeoylquinic acid) (**13**), *O*-caffeoylquinic acid (**16**), 3,4-di-*O*-caffeoylquinic acid (**38**), and 3,5-di-*O*-caffeoylquinic acid (**41**), corresponding to the caffeic acid part of the molecule. Discrimination between the isomers of di-*O*-caffeoylquinic acids was based on their MS^2^ fragmentation spectra, a base signal at *m*/*z* 173 for 4-acyl derivatives, at *m*/*z* 191 for 5-acyl derivatives, and at *m*/*z* 179 for 3-acyl derivatives, depending on the proton transfer, as previously reported [29]. 4,5-Di-*O*-caffeoylquinic acid (**43**) was also identified in the same way with base signal at *m*/*z* 173. The presence of the pseudo-molecular ion [M − H]^−^ at *m*/*z* 179 also allowed us to identify caffeic acid (**24**). Feruloyl-quinic acid derivatives were also detected with a pseudo-molecular ion [M − H]^−^ at *m/z* 367 for *O*-feruloyl-quinic acid (**26**) and at *m*/*z* 529 for caffeoyl-feruloyl-quinic acid derivatives (**46**, **47**). The flavonoids glycosides putatively identified were mostly various quercetin and kaempferol derivatives. Quercetin-*O*-pentosyl-hexosyl-hexoside (**25** [M − H]^−^ at *m*/*z* 741, [M-pentosyl − H]^−^ at *m*/*z* 609), rutin (**29**, [M − H]^−^ at *m*/*z* 609) and quercetin-*O*-(hydroxy-3-methylglutaryl)-hexoside (**36**, [M − H]^−^ at *m/z* 607, [M-hydroxy-3-methylglutaryl − H]^−^ at *m*/*z* 463) presented the characteristic fragment signal at *m*/*z* 301/300, corresponding to the quercetin aglycon (with a loss of one or two H) [30]. Moreover, kaempferol-*O*-pentosyl-hexosyl-hexoside (**28**, [M − H]^−^ at *m*/*z* 725, [M-pentosyl − H]^−^ at *m*/*z* 593), kaempferol-*O*-hexosyl-pentoside (**33**, [M − H]^−^ at *m*/*z* 593), kaempferol-3-*O*-rutinoside (**35**, [M − H]^−^ at *m*/*z* 593), kaempferol-*O*-hexoside (**37**, [M − H]^−^ at *m*/*z* 447), kaempferol-*O*-pentoside (**42**, [M − H]^−^ at *m*/*z* 417) were identified as kaempferol derivatives by the presence of the important signal at *m*/*z* 284/285, corresponding to the kaempferol aglycone (with a loss of one or two H). Glycolipid derivatives, mainly detected in ARE, were also putatively identified as two trihydroxy-dienoic acid derivatives (**51** and **52**, [M − H]^−^ at *m*/*z* 327) and a trihydroxy-octadecenoic acid derivative (**53**, [M − H]^−^ at *m*/*z* 329). Four hexosyl lipids were also possibly identified as *O*-(hexosyl-hexosyl)-*O*-linolenoyl-glycerol (**55**, [M − H]^−^ at *m*/*z* 675, [M-linolenoyl − H]^−^ at *m*/*z* 397), *O*-hexosyl-*O*-linolenoyl-glycerol (**57**, [M − H]^−^ at *m*/*z* 559, [M-hexosyl-glycerol − H]^−^ at *m*/*z* 277), *O*-(hexosyl-hexosyl)-*O*-palmitoyl-glycerol (**58**, [M − H]^−^ at *m*/*z* 699, [M-palmitoyl − H]^−^ at *m*/*z* 397) and *O*-hexosyl-di-*O*-linolenoyl-glycerol (**60**, [M − H]^−^ at *m*/*z* 559, [M-*O*-linolenoyl-*O*-hexosyl-glycerol − H]^−^ at *m*/*z* 277). In addition, the presence of two tyramines was also proposed: *N*-*trans*-*p*-coumaroyl-tyramine (**48**, [M − H]^−^ at *m*/*z* 282, and a fragment corresponding to coumaramide at *m*/*z* 162) and *N*-*trans*-feruloyl-tyramine (**49**, [M − H]^−^ at *m*/*z* 312, and a fragment corresponding to [ferulamide-CH_2_ − H]^−^ at *m*/*z* 178), mainly detected in ARE.

The presence of compounds **13**, **29**, **35**, **37**, **41**, **48**, and **49** was further supported by co-injection with commercial standards. To the best of our knowledge, nineteen phenolic compounds (**4**, **10**, **14**, **16**, **19**, **25**, **26**, **28**, **33**, **35**, **36**, **38**, **41**–**43**, and **46**–**49**) are hereby potentially characterized for the first time in *C. arvensis* by molecular networking. Resin glycosides and alkaloids were detected neither in ARE nor in ARM.

### 2.4. Quantification and Biological Screening of Major Compounds of ARE and ARM

**Figure 4:**
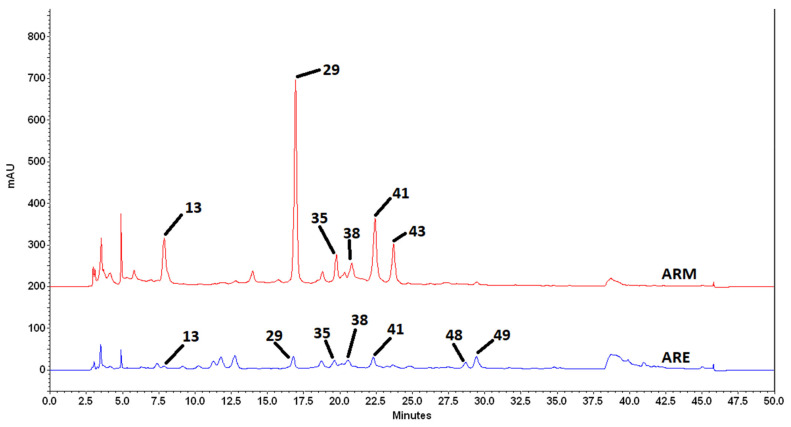
**UV chromatogram of ARE and ARM at 254 nm.** Chlorogenic acid (**13**), rutin (**29**), kaempferol-3-*O*-rutinoside (**35**), 3,4-di-*O*-caffeoylquinic acid (**38**), 3,5-di-*O*-caffeoylquinic acid (**41**), 4,5-di-*O*-caffeoylquinic acid (**43**), *N*-*trans*-*p*-coumaroyltyramine (**48**), and *N*-*trans*-feruloyltyramine (**49**).

To go one step further, the quantification analysis of main UV-absorbing constituents detected in both extracts was done by HPLC-PDA (Figure 4). Thus, chlorogenic acid (**13**), rutin (**29**), kaempferol-3-*O*-rutinoside (**35**), 3,5-di-*O*-caffeoylquinic acid (**41**), and *N*-*trans*-*p*-coumaroyltyramine (**48**) were quantified using their respective authentic standards (Table 2). 3,4-di-*O*-caffeoylquinic acid (**38**), 4,5-di-*O*-caffeoylquinic acid (**43**), and *N*-*trans*-feruloyltyramine (**49**) were quantified as equivalent of 3,5-di-*O*-caffeoylquinic acid (**41**) and *N*-*trans*-*p*-coumaroyltyramine (**48**), respectively, due to the lack of adequate standards quantities (Table 3). Chlorogenic acid (**13**), rutin (**29**), and 3,5-di-*O*-caffeoylquinic acid (**41**) were found as main constituents of ARM with the presence of 29.86 ± 0.17, 27.71 ± 0.19, and 38.16 ± 0.13 mg/g of dry extract, respectively. For ARE, the tyramines derivatives (**48**, **49**) and the 3,5-di-*O*-caffeoylquinic acid (**41**) were found as main constituents with amounts of 5.90 ± 0.27, 6.61 ± 0.40, and 5.82 ± 0.32 mg/g of dry extract, respectively. Sugars and derivatives, as well as lipids and glycolipids, not absorbing in UV, are not quantifiable by this method. Based on these amounts, we calculated the concentration of these compounds in the assay we performed on the J774 cells where we evaluated the effect of ARE and ARM on the expression of pro-inflammatory mediators in LPS-stimulated J744 cells (Table 2 and Table 3).

**Figure 5:**
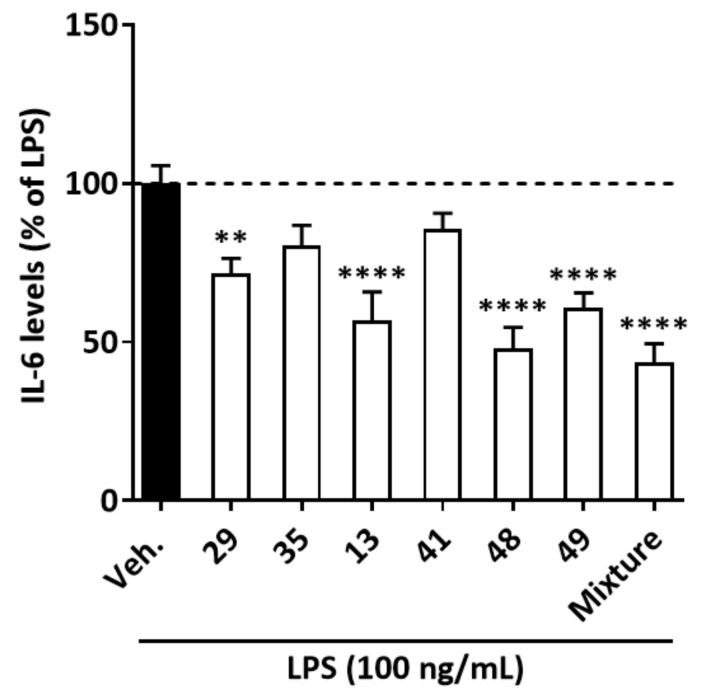
**Effect of ARE, ARM, standards, and the mixture of standards on the expression of IL-6 in LPS-stimulated J744 cells.** Cells were incubated with standards (5 µM), their mixture (same concentration of standards as in 50 µg/mL ARM), or vehicle (Veh., 0.25% DMSO) and LPS (100 ng/mL) for 8 h. IL-6 protein levels in the supernatant medium were quantified by ELISA. IL-6 was not detected in the medium of the unstimulated, vehicle-treated, cells. Results are expressed as percentage of the LPS-vehicle condition. The data were analyzed by the one-way ANOVA followed by Dunnet’s post hoc test for comparisons between groups, are expressed as mean ± SEM. *n* = 4 in triplicates, ** *p* < 0.05, **** *p* < 0.0001 vs. Veh. Rutin (**29**); kaempferol-3-*O*-rutinoside (**35**); chlorogenic acid (3-*O*-caffeoylquinic acid) (**13**); 3,5-di-*O*-caffeoylquinic acid (**41**); *N*-*trans*-*p*-coumaroyltyramine (**48**); *N*-*trans*-feruloyltyramine (**49**); mixture (**29**, **35**, **13**, **41**, **48**, **49**).

Next, to assess whether the main identified compounds of ARE and ARM could explain the activity of the extracts, rutin (**29**), kaempferol-3-*O*-rutinoside (**35**), chlorogenic acid (**13**), 3,5-di-*O*-caffeoylquinic acid (**41**), *N*-*trans*-*p*-coumaroyltyramine (48), and *N*-*trans*-feruloyltyramine (**49**), at 5 µM concentration, were tested on LPS-activated J774 cells. Additionally, a mixture of these compounds (**13**, **29**, **35**, **41**, **48**, **49**) with the same concentration as in 50 µg/mL ARM was also tested to evaluate if they have some additive or synergistic effect against IL-6 production and can explain the activity observed (Figure 5). In our hands, rutin (**29**), chlorogenic acid (**13**), *N*-*trans*-*p*-coumaroyltyramine (**48**), and *N*-*trans*-feruloyltyramine (**49**) were the most active, with IL-6 levels representing 71.6 ± 4.8, 56.8 ± 8.9, 48 ± 6.6, and 60.9 ± 4.6%, respectively, of those found in the presence of vehicle. In the mixture, IL-6 levels were 43.6 ± 5.8% compared to vehicle.

This is the first reported effect of *N*-*trans*-*p*-coumaroyltyramine (**48**) on pro-inflammatory cytokine production. Rutin (**29**) is an important dietary flavonoid with several therapeutic effects [42]. It was found to dose dependently (25, 50, and 100 µM) suppress the activation of NF-κB and production of TNF-α in LPS-activated primary human umbilical vein endothelial cells (HUVECs) [43]. Chlorogenic acid (**13**) is an important biologically active phenolic compound with several therapeutic activities [44]. At 20 µM concentration, it was found to decrease iNOS-mediated nitric oxide production, and to decrease pro-inflammatory cytokines (e.g., IL-1β, TNF-α, IL-6, and CXCL1) expression through down-regulation of NF-κB in LPS-stimulated RAW 264.7 macrophages [45]. Results obtained in our study show a significant effect of chlorogenic acid (**13**) on IL-6 production at 5 µM. *N*-*trans*-feruloyltyramine (**49**) (tested at 160 µM) was found to significantly decrease NO, PGE_2_, and ROS production and down-regulate iNOS and COX-2 mRNA expression in LPS-stimulated RAW 264.7 cells. These effects were associated with inactivation of AP-1 and MAPKs, which resulted from blocking of JNK phosphorylation [46]. Our data show a significant effect of rutin (**29**), chlorogenic acid (**13**), and *N*-*trans*-feruloyltyramine (**49**) on LPS-induced IL-6 production at 5 µM.

Moreover, the levels of IL-6 production (as % of LPS-vehicle condition) in ARM-treated and the mixture-treated cells are 27.3 ± 3.8% (data taken from Figure 2F) and 43.6 ± 5.8% (data taken from Figure 5), respectively. The stronger inhibitory effect of ARM on IL-6 production compared to the mixture suggests that, besides the most abundant ones, other compounds present in the ARM extract also contribute to the activity.

Chlorogenic acid (**13**), rutin (**29**), and tyramine derivatives (**48**, **49**) are water-soluble compounds, but the decoction (ARW) did not inhibit the expression of IL-6 in our LPS-activated J774 cells model. In our HPLC-PDA analysis of ARW, we did not detect tyramine derivatives (**48**, **49**), which may be degraded during boiling in water. Chlorogenic acid (**13**) and rutin (**29**) were detected and quantified as 28.23 ± 0.86 and 13.87 ± 0.07 mg/g of dry extract, respectively. Two hypotheses could explain the absence of activity of ARW despite the presence of chlorogenic acid (**13**) and rutin (**29**). Firstly, **13**, **29**, **48**, and **49** are not the only active compounds of ARE and ARM, rather there are also other compounds contributing to the anti-inflammatory activity of ARE and ARM. Secondly, ARW may contain some compounds which are antagonizing the effect of active compounds.

Finally, from a mechanistic perspective, as the crude extracts contain different chemical constituents and act by several synergistic mechanisms, further studies are required to study the effect of ARE and ARM on different inflammatory pathways. These could include NF-κB and MAPK pathways as chlorogenic acid **(13)**, rutin (**29**), and *N*-*trans*-feruloyltyramine (**49**) have already been reported to inhibit these pathways in LPS-activated macrophage in vitro models.

## 3. Materials and Methods

### 3.1. Chemicals and Reagents

HPLC grade acetonitrile, ethyl acetate, dichloromethane, hexane, and methanol were purchased from VWR International (Radnor, PA, USA). Kaempferol-3-*O*-rutinoside (**35**) and 3,5-di-*O*-caffeoylquinic acid (**41**) were purchased from Extrasynthèse (Genay, France). *N*-*trans*-*p*-coumaroyltyramine (**48**) was purchased from Phytolab GmbH & Co. KG (Vestenbergsgreuth, Germany). Chlorogenic acid (3-*O*-caffeoylquinic acid) (**13**), rutin (**29**), *N*-*trans*-feruloyltyramine (moupinamide) (**49**), dimethylsulfoxide (DMSO), camptothecin, (3-(4,5-dimethylthiazol-2-yl)-2,5-diphenyltetrazolium bromide (MTT), fetal bovine serum (FBS), and lipopolysaccharides (LPS from *E. coli* serotype O55:B5) were purchased from Sigma-Aldrich (Bornem, Belgium).

### 3.2. Collection of Plant

*C. arvensis* whole plant (aerial parts, roots, and flowers) was collected from the crop fields of Mouza As-haba, Jhang, Province Punjab, Pakistan in the month of April–May 2018. The collected plant material was authenticated by Dr. Zafarullah Zafar, Institute of Pure and Applied Biology/Botany Division, Bahauddin Zakariya University Multan, Pakistan. A voucher number (R. R. Stewart, F. W. Pak. 572(2) at BZU Pakistan and PAK-ZAFAR 002 at GNOS UCLouvain) was assigned for future reference. The plant name was further confirmed by checking in The Plant List (http://www.theplantlist.org, accessed on 26 October 2019).

### 3.3. Preparation of Crude Extracts

The collected *C. arvensis* whole plants were washed with tap water, shade dried and then ground. Then, 50 g of the powdered plant material was extracted in a Soxhlet apparatus for 8 h by using successively 250 mL of hexane, dichloromethane, ethyl acetate, and methanol. The solvents were removed by rotary evaporator and four corresponding crude extracts (ARH, ARD, ARE, and ARM, respectively) were obtained. A decoction (ARW) was also prepared by boiling 50 g of the plant material in 1 L of water for 15 min, filtered hot, and water was removed by lyophilization. The extracts and the decoction were stored at −20 °C until further use.

### 3.4. Cell Cultures

Murine macrophage cell line J774 (a kind gift from Prof Van Bambeke, LDRI, UCLouvain) and human lung fibroblast cell line WI38 (ATCC CCL-75, bought from LGC standards, Molsheim, France) were grown in Roswell Park Memorial Institute medium (RPMI 1640 medium with GlutaMAX, Gibco, Thermo Fisher Scientific^®^, Bleiswijk, The Netherlands) and Dulbecco’s modified eagle medium (DMEM, containing 1 g/L glucose and 1 mM pyruvate, Gibco), respectively, supplemented with 10% fetal bovine serum (FBS) and penicillin-streptomycin (100 UI/mL) (Lonza, Verviers, Belgium), maintained at 37 °C in 5% CO_2_ incubator.

### 3.5. MTT Assay

The crude extracts were analyzed by MTT assay on J774 and WI38 cells. Cells were seeded overnight in a 96-well plate at a density of 5 × 10^3^ cells per well in 180 μL per well of their respective culture medium. After 24 h, cells were treated with stock solutions of crude extracts, diluted in the respective culture medium, 20 µL per well, with the final concentration of 100 µg/mL and 50 µg/mL, and incubated for further 72 h. Based on their solubility, stock solutions of ARH, ARD, ARE, and ARM were prepared in DMSO while those of ARW in EtOH-H_2_O (25:75). At the end of the incubation, medium was removed and the cells were incubated for 45 min with 100 µL of MTT solution prepared by dissolving 15 mg of MTT in 5 mL of PBS and 45 mL of the respective culture medium. Next, MTT solution was replaced by DMSO (100 µL per well) and the absorbance was measured with a spectrophotometer (SpectraMax-Molecular Devices, Berkshire, UK) at 570 nm (with 620 nm reference wavelength) [47]. Camptothecin was used as a positive control while medium with vehicles (DMSO and EtOH-H_2_O (25:75)) at 0.5% were used as negative controls. All experiments were performed at least two times in triplicate.

### 3.6. Effect of Crude Extracts and Identified Major Compounds on the Expression of Pro-Inflammatory Mediators in LPS-Stimulated J774 Cells

J774 cells were seeded overnight in 1 mL per well of RPMI medium at the density of 2.5 × 10^5^ cells/well in a 24-well plate. Then, the medium was removed and the cells were treated with the crude extracts solutions (50 µg/mL), or vehicle (0.25% DMSO) with or without LPS (100 ng/mL) [48]. Identified main compounds of the extracts were tested at 5 µM concentration and a mixture of them at the same concentration as in 50 µg/mL ARM. After 8 h incubation, supernatants were collected and stored at −20 °C for ELISA and cell culture plates were stored at −80 °C.

### 3.7. Real-Time Quantitative PCR (qPCR)

Total RNA from the cells was extracted using TriPure reagent (Roche, Basel, Switzerland) according to the manufacturer’s instructions. cDNA was synthesized from 1 µg of total RNA using a reverse transcription kit (RT GoScript kit, Promega Benelux BV, Leiden, The Netherlands). Real-time qPCR analysis was performed on a QuantStudio 3 instrument (Applied Biosystems, Thermo Fisher Scientific^®^, Bleiswijk, The Netherlands) using a SYBR Green mix (GoTaq qPCR Master mix, Promega). The following conditions were used for amplification: an initial holding stage of 10 min at 95 °C, then 45 cycles consisting of denaturation at 95 °C for 3 s, annealing at 60 °C for 26 s, and extension at 72 °C for 10 s. At the end of the PCR reaction, melting curves of the products were obtained. The resulting cycle threshold (Ct) were recorded for each gene and normalized using 60S ribosomal protein L19 (RPL19) mRNA as reference. Results are expressed relative to control, using the “delta-delta Ct” method. Primer sequences are given in Table 4 [49].

### 3.8. Cytokines Quantification by ELISA

Concentrations of IL-6, TNF-α, and MCP-1 in the collected cell culture supernatants were determined by a sandwich type ELISA technique using the Ready-Set-Go! Kit following the manufacturer’s instructions (Invitrogen, Thermo Fisher Scientific^®^, Bleiswijk, The Netherlands) [50].

### 3.9. HPLC-PDA Analysis

Chromatographic separation was performed on an HPLC-PDA system consisting of a Thermo Accela pump and PDA ray detector (Thermo Scientific^TM^, Bremen, Germany). The LC separation was done on a Phenomenex Luna C18, 250 × 4.6 mm packed with 5 µm particles. Stock solutions of standards were prepared at 1 mg/mL concentration in HPLC grade methanol and then serially diluted to achieve five different concentrations in the range of: 500−5 µg/mL for **29**; 200−5 µg/mL for **41**; 50−1 for **48**; and 100−1 µg/mL for **13** and **35**. All analyses were carried out with 20 µL injection volume and with a flow rate of 0.8 mL/min. Detection wavelength was set between 200 to 400 nm. The mobile phase consisted of 0.1% of formic acid in water (solvent A) and 100% acetonitrile (solvent B). Elution of the mobile phase was performed in gradient mode: 0–5 min (17% B); 5–35 min (17–40% B); 35–36 min (40–100% B); 36–42 min (100% B); 42–43 min (100–17% B); and 43–50 min (17% B). Chromatograms were integrated at 355 nm for **29** and **35**; 290 nm for **48**; and at 327 nm for **13** and **41**. ARE and ARM solutions were freshly prepared at 8 mg/mL in HPLC grade methanol and analyzed under the same conditions as standards. LOD and LOQ were calculated from the residual standard deviation (σ) of the regression curves and the slopes (S), according to the following equations: LOD = 3.3 σ/S and LOQ = 10 σ/S.

### 3.10. HPLC-DAD-HRMS/MS Analysis

HPLC separations were conducted as described previously in HPLC-PDA analysis, and connected with a Thermo Scientific LTQ orbitrap XL mass spectrometer (Thermo Scientific^TM^, Bremen, Germany). The instrument was controlled using Thermo Scientific Xcalibur X software. The LC separation was done on a Phenomenex Luna C18, 250 × 4.6 mm packed with 5µm particles. All analyses were carried out with 20 µL injection volume, with the flow rate of 0.8 mL/min, and with the mobile phase consisting of 0.1% of formic acid in water (solvent A) and 100% acetonitrile (solvent B). Elution of the mobile phase was performed in gradient mode: 0–5 min (17% B); 5–27 min (17–40% B); 27–28 min (40–100% B); 28–38 min (100% B); 38–39 min (100–17% B); and 39–50 min (17% B). Chromatograms were recorded between 200 and 600 nm. HRMS analyses were realized in APCI negative and positive modes with the following inlet conditions: for negative mode: capillary temperature 250 °C; APCI vaporizer temperature 400 °C; sheath gas flow 25 a.u.; auxiliary gas flow 25 a.u. and sweep gas flow 5 a.u. Discharge current of 5 µA; capillary temperature of 250 °C; capillary voltage of −10 V and tube lens voltage of −125 V. For positive mode: capillary temperature 250 °C; APCI vaporizer temperature 400 °C; sheath gas flow 25 a.u.; auxiliary gas flow 25 a.u. and sweep gas flow 5 a.u.; discharge current of 5 µA; capillary temperature of 250 °C; capillary voltage of 21 V and tube lens voltage of 75 V. The data-dependent MS/MS events were performed on the three most intense ions detected in full scans MS.

### 3.11. MS Data Treatment

All HRMS run data (.RAW) files were exported to the open source software package MZmine 2 (2.53 version) for data processing [51]. For mass detection at MS^1^ level, the noise level was set to 1.0 × 10^5^ for negative mode (APCI source) (negative mode was found to be more informative than positive mode). For MS^2^ detection, the noise level was set to 0. The ADAP chromatogram builder was used and set to a minimum group size of scans of 4, a minimum group intensity of 1.0 × 10^4^, a minimum highest intensity of 1.0 × 10^5^, and *m*/*z* tolerance of 5 ppm. The ADAP algorithm (wavelets) was used for chromatogram deconvolution. The intensity window S/N was used as an S/N estimator with a signal to noise ratio set at 8, a minimum feature height of 1.0 × 10^5^, a coefficient area threshold at 10, a peak duration ranging from 0.02 to 0.8 min, and the RT wavelet range from 0.02 to 0.2 min. Isotopes were detected using the isotope peak grouper with a *m/z* tolerance of 5 ppm, a RT tolerance of 0.02 min (absolute), the maximum charge set at 1, and the representative isotope used was the most intense. Then, the aligned list peak was gap-filled with RT range of 0.04 min and *m*/*z* tolerance of 8 ppm. The resulting list was filtered using the peak list rows filter option to remove all the duplicates and all the features without MS^2^ spectrum associated.

### 3.12. Mass Spectral Organization and Dereplication

A molecular network was constructed from the .mgf file exported from MZmine, using the online workflow on the GNPS website [22]. The precursor ion mass tolerance was set to 0.02 Da with a MS/MS fragment ion tolerance of 0.02 Da. A network was then created where edges were filtered to have a cosine of 0.7 and more than four matched peaks. The spectra in the network were then searched against GNPS’s spectral libraries filtered under the same conditions as before. All matches kept between network spectra and library spectra were required to have a score of 0.75 and at least three matched peaks. Additional putative identification of unmatched peaks was carried out comparing available MS/MS fragmentation patterns in the literature. Data visualization was achieved using Cytoscape 3.8.0 [52]. Peak area data from the .csv file obtained from MZmine was added to the network. Size nodes were set proportionally to the total area of each peak detected in both analyzed extracts. Edge widths were set corresponding to the cosine score.

## 4. Conclusions

We report that ethyl acetate (ARE) and methanol (ARM) extracts of *C. arvensis* significantly inhibited the expression of pro-inflammatory mediators by activated J774 cells. Using a dereplication strategy, 45 compounds were putatively identified, among which rutin, kaempferol-3-*O*-rutinoside, chlorogenic acid (3-*O*-caffeoylquinic acid), 3,5-di-*O*-caffeoylquinic acid, *N*-*trans*-*p*-coumaroyltyramine, and *N*-*trans*-feruloyltyramine were among the major compounds present in both active extracts. These compounds were quantified and tested on LPS-activated J774 cells where they were shown to be responsible for a part of the observed effects against IL-6 production. Taken together, our studies will contribute to a better understanding of the chemical composition and the biological properties of *Convolvulus arvensis*.

## Figures and Tables

**Table 1:** Putative identification of chemical constituents present in *C. arvensis* extracts (ARE and ARM) by comparison of the MS^2^ data (Negative mode, APCI).

Code	t_R_ [min]	λ_max_	*m*/*z*	MS Major Ion(s)	MS/MS Fragments [*m*/*z*]	Molecular Formula	Δ ppm	Δ mDa	Putative Identification	Isolated Previously ^a^	Ref.
**1**	3.20	n.d.	387.1147	[M + HCOO^−^]^−^	179.0563 ^b^	C_12_H_22_O_11_	0.04	0.01	Sucrose		
			341.1084	[M − H]^−^							
			729.2287	[2M + HCOO^−^]^−^							
			683.2222	[2M − H]^−^							
**2**	3.28	n.d.	151.0613	[M − H]^−^	71.0142; 101.0247; 133.0509 ^b^	C_5_H_12_O_5_	4.31	0.65	Xylitol ^#^		
			197.0664	[M + HCOO^−^]^−^							
**3**	3.40	n.d.	181.0722	[M + HCOO^−^]^−^	135.0669 ^c^	C_5_H_12_O_4_	−2.01	−0.27	2-methyl-1,2,3,4-butanetetrol ^#^		
			135.0668	[M − H]^−^							
**4**	3.39	270	191.0567	[M − H]^−^	85.0299; 127.0405; 173.0458; 93.0350 ^b^	C_7_H_12_O_6_	5.95	1.14	Quinic acid ^#^	*C. althaeoides*	[31]
			383.1199	[2M − H]^−^							
**5**	3.59	n.d.	239.0775	[M + HCOO^−^]^−^	133.0509; 59.0141 ^b^	C_7_H_14_O_6_	5.93	1.09	*O*-methyl-inositol isomer I ^#^		
			193.0723	[M − H]^−^							
**6**	3.96	n.d.	239.0772	[M + HCOO]^−^	193.0718; 133.0508; 59.0141 ^b^	C_7_H_14_O_6_	2.12	0.51	*O*-methyl-inositol isomer II ^#^		
**7**	4.90	n.d.	239.0772	[M + HCOO^−^]^−^	133.0508; 59.0141 ^b^	C_7_H_14_O_6_	3.04	0.59	*O*-methyl-inositol isomer III ^#^		
			193.0718	[M − H]^−^							
			712.5355	[M − H]^−^							
**8**	4.98	n.d.	117.0198	[M − H]^−^	73.0297; 99.0091 ^b^	C_4_H_6_O_4_	8.69	1.02	Succinic acid ^#^		
**9**	5.06	n.d.	281.0881	[M − H]^−^	235.0820; 263.0955 ^b^	C_10_H_18_O_9_	3.00	0.84	Xylobiose ^#^		
**10**	5.68	n.d.	341.0889	[M − H]^−^	179.0348; 167.0349; 135.0451 ^b^	C_15_H_18_O_9_	4.82	1.64	*O*-glucosyl-caffeic acid isomer ^#^		
**11**	6.02	n.d.	451.2199	[M + HCOO^−^]^−^	167.1076; 179.0560; 243.1596 ^b^	C_19_H_34_O_9_	−3.60	−1.46	Magastigmane glycoside derivative I ^#^		
			405.2110	[M − H]^−^							
**12**	6.18	289; 325	433.2091	[M + HCOO^−^]^−^	387.2012; 179.0559; 161.0454 ^c^	C_19_H_32_O_8_	3.99	1.73	Magastigmane glycoside derivative II ^#^		
**13**	6.21	n.d.	353.0881	[M − H]^−^	191.0559; 179.0350; 173.0455; 135.0452 ^b^	C_16_H_18_O_9_	2.39	0.84	Chlorogenic acid *	*C. arvensis; C. dorycnium*	[32,33]
**14**	7.30	315	369.0820	[M + HCOO^−^]^−^	323.0769; 161.0243 ^b^	C_15_H_16_O_8_	0.64	0.21	Skimmin ^#^	*Pharbitis nil*	[34]
			323.0769	[M − H]^−^							
**15**	7.97	n.d.	395.1930	[M + HCOO^−^]^−^	187.1341; 161.0457; 179.0563 ^b^	C_16_H_30_O_8_	5.03	1.76	Monoterpenoid glycoside I ^#^		
			349.1880	[M − H]^−^							
**16**	8.66	245; 324	353.0881	[M − H]^−^	191.0560; 179.0350 ^b^	C_16_H_18_O_9_	2.39	0.84	*O*-caffeoylquinic acid ^#^	*Ipomoea batatas*	[35]
			707.1868	[2M − H]^−^							
**17**	9.06	288	433.2091	[M + HCOO^−^]^−^	161.0455 ^b^	C_19_H_32_O_8_	−2.88	−1.12	Magastigmane glycoside derivative III ^#^		
			387.2014	[M − H]^−^							
**18**	10.20	287	387.1870	[M − H]^−^	207.1022; 163.1128; 369.1544 ^b^	C_15_H_32_O_11_	0.94	0.36	n.i.		
**19**	10.51	n.d.	431.1928	[M + HCOO^−^]^−^	223.1337; 205.1233; 153.0922; 161.0457 ^b^	C_19_H_30_O_8_	1.97	0.76	Roseoside ^#^	*Ipomoea purpurea*	[36]
			385.1853	[M − H]^−^							
**20**	11.36	322	297.0986	[M − H]^−^	179.0352; 135.0453 ^b^	C_14_H_18_O_7_	3.95	1.17	n.i.		
**21**	12.13	265	441.1969	[M + HCOO^−^]^−^	n.s.	C_17_H_32_O_10_	−4.61	−1.82	n.i.		
			395.1899	[M − H]^−^							
**22**	12.28	n.d.	281.1397	[M − H]^−^	237.1488; 171.1180; 123.0817; 201.1284 ^b^	C_15_H_22_O_5_	2.85	0.80	n.i.		
**23**	12.97	258	583.2027	[M + HCOO^−^]^−^	n.s.	C_26_H_34_O_12_	−1.49	−0.80	n.i.		
			537.1964	[M − H]^−^							
			1187.3107	[2M − H]^−^							
			1613.8181	[M − H]^−^							
**24**	13.26	242; 298; 324	179.0353	[M − H]^−^	135.0453 ^b^	C_9_H_8_O_4_	4.84	0.87	Caffeic acid ^#^	*C. trabutianus*	[37]
**25**	14.25	255; 331	741.1902	[M − H]^−^	300.0269; 609.1447; 301.0347; 591.1343; 271.0242 ^b^	C_32_H_38_O_20_	3.21	2.38	Quercetin-*O*-pentosyl-hexosyl-hexoside ^#^		
**26**	14.33	294; 326	367.1024	[M − H]^−^	191.0559; 173.0455; 193.0499 ^b^	C_17_H_20_O_9_	−1.38	−0.51	Feruloyl quinic acid ^#^		
**27**	14.78	n.d.	225.1138	[M − H]^−^	181.1234; 165.0921; 147.0816; 135.0816 ^b^	C_12_H_18_O_4_	4.96	1.12	Tuberonic acid ^#^	*Dichondra repens*	[38]
**28**	16.09	n.d.	725.1935	[M − H]^−^	593.1494; 575.1391; 284.0317; 285.0394; 327.0500 ^b^	C_32_H_38_O_19_	0.82	0.60	Kaempferol-*O*-pentosyl-hexosyl-hexoside ^#^		
**29**	16.54	254; 347	609.1460	[M − H]^−^	301.0349; 300.0276; 271.0248; 343.0454 ^b^	C_27_H_30_O_16_	0.72	0.44	Rutin *	*C. arvensis; Cressa cretica*	[15,39]
			1219.3008	[2M − H]^−^							
**30**	16.78	n.d.	621.3154	[M + HCOO^−^]^−^	557.2985 ^b^	C_28_H_48_O_12_	3.91	2.25	n.i.		
			575.3090	[M − H]^−^							
**31**	17.24	n.d.	665.3405	[M + HCOO^−^]^−^	619.3352; 601.3245 ^c^	C_31_H_54_O_15_	3.61	2.23	n.i.		
**32**	17.84	n.d.	709.3664	[M + HCOO^−^]^−^	645.3510; 663.3615 ^c^	C_32_H_56_O_14_	2.45	1.74	n.i.		
**33**	18.00	240; 301; 325	593.1503	[M − H]^−^	285.0398; 284.0325; 327.0507; 255.0297 ^b^	C_27_H_30_O_15_	−0.58	−0.35	Kaempferol-*O*-hexosyl-pentoside ^#^		
**34**	18.36	309	753.3912	[M + HCOO^−^]^−^	689.3768; 707.3875 ^c^	C_34_H_60_O_15_	0.43	0.32	n.i.		
**35**	18.73	238; 265; 342	593.1503	[M − H]^−^	285.0369; 327.0500 ^b^	C_27_H_30_O_15_	−0.58	−0.35	Kaempferol-3-*O*-rutinoside *^#^	*C. dorycnium*	[40]
			1187.3107	[2M − H]^−^							
**36**	19.20	297; 342	607.1292	[M − H]^−^	463.0877; 505.0982; 545.1293; 301.0352 ^b^	C_27_H_28_O_16_	−1.17	−0.71	Quercetin-*O*-[-*O*-(hydroxy-3-methylglutaryl)-hexoside] ^#^		
**37**	19.56	301; 325	447.0950	[M − H]^−^	284.0324; 285.0399; 327.0507; 255.0297 ^b^	C_21_H_20_O_11_	5.06	2.26	Kaempferol-3-*O*-glucoside *^#^	*C. trabutianus*	[37]
**38**	19.69	238; 325	515.1215	[M − H]^−^	353.0858; 173.0454; 335.0765; 179.0347 ^b^	C_25_H_24_O_12_	4.95	2.55	3,4-di-*O*-caffeoylquinic acid ^#^	*C. trabutianus*	[37]
			1031.2482	[2M − H]^−^							
**39**	20.06	n.d.	511.2174	[M + HCOO^−^]^−^	161.0245; 179.0350 ^c^	C_24_H_34_O_9_	−1.05	−0.54	n.i.		
			929.4974	[2M − H]^−^							
**40**	20.52	n.d.	973.5257	[M − H]^−^	909.508 ^b^	C_46_H_78_N_4_O_18_	2.48	2.41	n.i.		
**41**	20.96	245; 295; 327	515.1211	[M − H]^−^	353.0868; 191.056; 179.0349 ^b^	C_25_H_24_O_12_	4.17	2.15	3,5-di-*O*-caffeoylquinic acid *^#^	*C. trabutianus*	[37]
			561.1236	[M + HCOO^−^]^−^							
			1031.2471	[2M − H]^−^							
**42**	21.67	n.d.	417.0831	[M − H]^−^	284.0323; 285.0397; 327.0506; 255.0295 ^b^	C_20_H_18_O_10_	2.23	1.80	Kaempferol-*O*-pentoside ^#^		
**43**	21.86	245; 293; 324	515.1214	[M − H]^−^	353.0866; 299.056; 173.0457; 203.0350; 191.0560 ^b^	C_25_H_24_O_12_	2.37	0.93	4,5-di-*O*-caffeoylquinic acid ^#^		
			1031.2463	[2M − H]^−^							
**44**	22.49	n.d.	187.0981	[M − H]^−^	125.0975; 97.0663 ^b^	C_9_H_16_O_4_	5.70	1.07	Azelaic acid ^#^		
**45**	22.93	n.d.	625.2126	[M + HCOO^−^]^−^	n.s.	C_28_H_36_O_13_	−0.98	−0.57	n.i.		
			579.2072	[M − H]^−^							
**46**	24.91	n.d.	529.1365	[M − H]^−^	353.0868; 367.1023; 191.0561 ^b^	C_26_H_26_O_12_	3.59	1.90	Caffeoyl-feruloyl quinic acid isomer I ^#^		
**47**	25.41	n.d.	529.1372	[M − H]^−^	367.1016; 173.0454; 193.0506; 179.0349 ^b^	C_26_H_26_O_12_	4.91	2.60	Caffeoyl-feruloyl quinic acid isomer II ^#^		
**48**	26.09	292; 305	282.1146	[M − H]^−^	119.0506; 145.0296; 162.0563 ^b^	C_17_H_17_NO_3_	5.61	1.58	*N*-*trans*-*p*-coumaroyltyramine *^#^	*Ipomoea batatas*	[41]
			328.1194	[M + HCOO^−^]^−^							
**49**	26.67	238; 293; 318	312.1243	[M − H]^−^	178.0512; 297.1007; 135.0454 ^b^	C_18_H_19_NO_4_	2.30	0.72	*N*-*trans*-feruloyltyramine *^#^	*Ipomoea batatas*	[41]
			358.1294	[M + HCOO^−^]^−^							
**50**	28.04	n.d.	607.2026	[M + HCOO^−^]^−^	561.1960 ^c^	C_28_H_34_O_12_	−0.13	−0.08	n.i.		
**51**	29.36	n.d.	327.2182	[M − H]^−^	229.1438; 291.1964, 211.1336; 171.1029 ^b^	C_18_H_32_O_5_	3.21	1.05	Trihydroxy-10,15-octadecadienoic acid derivative I ^#^		
**52**	30.56	n.d.	327.2178	[M − H]^−^	229.1437; 291.1961; 171.1027; 211.1336; 309.2067 ^b^	C_18_H_32_O_5_	1.99	0.65	Trihydroxy-10,15-octadecadienoic acid derivative II ^#^		
**53**	31.36	n.d.	329.2329	[M − H]^−^	329.2333; 229.1440; 211.1337; 171.1030; 311.2228; 293.2122 ^b^	C_18_H_34_O_5_	0.31	0.10	Trihydroxy-10-octadecenoic acid ^#^		
**54**	31.90	n.d.	883.4227	[M + HCOO^−^]^−^	561.6332; 533.2184 ^b^	C_39_H_66_O_19_	2.62	2.20	*O*-(Hexosyl-hexosyl-hexosyl)-octadecatrienoyl-glycerol ^#^		
			837.4142	[M − H]^−^							
**55**	32.54	n.d.	721.3635	[M + HCOO^−^]^−^	397.1340 ^b^	C_33_H_56_O_14_	−0.86	−0.58	*O*-(Hexosyl-hexosyl)-*O*-linolenoyl-glycerol ^#^		
			675.3586	[M − H]^−^							
			712.5355	[M − H]^−^							
**56**	33.05	n.d.	647.3270	[M + HCOO^−^]^−^	n.s.	C_30_H_50_O_12_	−1.67	−1.00	n.i.		
			601.3214	[M − H]^−^							
**57**	33.53	218	559.3091	[M + HCOO^−^]^−^	277.2170; 253.0926 ^c^	C_27_H_46_O_9_	−4.89	−2.74	*O*-Hexosyl-*O*-linolenoyl-glycerol ^#^		
**58**	34.56	215	699.3825	[M + HCOO]^−^	397.1340 ^c^	C_31_H_58_O_14_	3.13	2.19	*O*-(Hexosyl-hexosyl)-*O*-palmitoyl-glycerol ^#^		
**59**	35.20	n.d.	1659.8471	[M + HCOO^−^]^−^	n.s.	C_98_H_118_O_20_	2.65	4.28	n.i.		
			1613.8181	[M − H]^−^							
**60**	35.87	218	819.5250	[M + HCOO^−^]^−^	773.5198; 277.2170; 513.3065 ^b^	C_45_H_74_O_10_	−0.74	−0.57	*O*-Hexosyl-di-*O*-linolenoyl-glycerol ^#^		
			773.5198	[M − H]^−^							
**61**	38.25	409	997.5783	[M + HCOO^−^]^−^	n.s.	C_51_H_84_O_16_	4.40	4.19	n.i.		
			951.5723	[M − H]^−^							
**62**	38.70	218	765.5196	[M − H]^−^	505.3003; 255.2320; 277.2163; 527.2846; 747.5024 ^b^	C_43_H_74_O_11_	5.63	4.31	n.i.		
**63**	40.11	409	591.2607	[M − H]^−^	559.2329; 515.2441 ^b^	C_34_H_40_O_9_	2.19	1.29	n.i.		
**64**	40.69	n.d.	1835.8597	[M − H]^−^	n.s.	C_116_H_124_O_20_	−0.58	−1.07	n.i.		
**65**	41.44	n.d.	758.5413	[M + HCOO^−^]^−^	n.s.	C_41_H_71_N_5_O_5_	−3.08	−2.20	n.i.		
			712.5355	[M − H]^−^							

^a^ Previously isolated from Convolvulaceae family species; ^b^ fragments produced from [M − H]^−^ adduct; ^c^ fragments produced from [M + HCOO^−^]^−^ adduct; n.d., not detectable by overlapping with near chromatographic compounds; n.s., no significant signals; n.i., no identified compound; * compounds identified by standard comparison. ^#^ compounds identified in *C. arvensis* for the first time.

**Table 2:** Major phenolic compounds quantified in ARE and ARM.

ID	Compound	Regression Equation	R^2^	LOD (µg/mL)	LOQ (µg/mL)	ARE			ARM		
						Conc. in Crude Extract (mg/g Dry Extract)	Concentration in 50 µg of Extract/mL		Conc. in Crude Extract (mg/g Dry Extract)	Concentration in 50 µg of Extract/mL	
							(µg/mL)	(µM)		(µg/mL)	(µM)
**13**	Chlorogenic acid	y = 40320x + 99146	0.9996	4.37	13.24	1.51 ± 0.04	0.08 ± 0.00	0.21 ± 0.01	29.86 ± 0.17	1.49 ± 0.01	4.22 ± 0.02
**29**	Rutin	y = 26787x + 147669	0.9997	5.11	15.48	0.78 ± 0.19	0.04 ± 0.01	0.06 ± 0.02	27.71 ± 0.19	1.39 ± 0.01	2.27 ± 0.02
**35**	Kaempferol-3-*O*-rutinoside	y = 36219x + 52756	0.9996	4.16	12.60	2.18 ± 0.02	0.11 ± 0.00	0.18 ± 0.00	5.23 ± 0.19	0.26 ± 0.01	0.44 ± 0.02
**41**	3,5-di-*O*-caffeoylquinic acid	y = 59073x + 308772	0.9971	8.68	26.32	5.82 ± 0.32	0.29 ± 0.02	0.56 ± 0.03	38.16 ± 0.13	1.91 ± 0.01	3.70 ± 0.01
**48**	*N*-*trans*-*p*-coumaroyltyramine	y = 88859x + 21246	0.9997	1.81	5.47	5.90 ± 0.27	0.30 ± 0.01	1.04 ± 0.05	1.72 ± 0.13	0.09 ± 0.01	0.30 ± 0.02

**Table 3:** Relative content of major phenolic compounds of ARE and ARM, expressed as: ^a^ mg of 3,5-di-*O*-caffeoylquinic acid equivalent; ^b^ mg of *N*-*trans*-*p*-coumaroyltyramine equivalent.

ID	Compound	ARE			ARM		
		Conc. in Crude Extract (mg/g Dry Extract)	Concentration in 50 µg of Extract/mL		Conc. in Crude Extract (mg/g Dry Extract)	Concentration in 50 µg of Extract/mL	
			(µg/mL)	(µM)		(µg/mL)	(µM)
**38**	3,4-di-*O*-caffeoylquinic acid	1.48 ± 0.04 ^a^	0.07 ± 0.00 ^a^	0.14 ± 0.00 ^a^	14.08 ± 0.81^a^	0.704 ± 0.04 ^a^	1.36 ± 0.08 ^a^
**43**	4,5-di-*O*-caffeoylquinic acid	1.97 ± 0.03 ^a^	0.10 ± 0.00 ^a^	0.19 ± 0.00 ^a^	21.34 ± 0.06 ^a^	1.067 ± 0.00 ^a^	2.07 ± 0.01 ^a^
**49**	*N*-*trans*-feruloyltyramine	6.61 ± 0.40 ^b^	0.33 ± 0.02 ^b^	1.06 ± 0.06 ^b^	1.37 ± 0.30 ^b^	0.068 ± 0.02 ^b^	0.22 ± 0.05 ^b^

**Table 4:** Primer sequences for qPCR.

Gene/Product	Forward Primer (5′ to 3′)	Reverse Primer (5′ to 3′)
*Il6*/IL-6	ACAAGTCGGAGGCTTAATTACACAT	TTGCCATTGCACAACTCTTTTC
*Tnf*/TNF-α	CTACTGAACTTCGGGGTGATC	TGAGTGTGAGGGTCTGGGC
*Ccl2*/MCP-1	GTCCCAAAGAAGCTGTAGTTTTTG	ATGTATGTCTGGACCCATTCC
*Rpl19*/RPL19	TGACCTGGATGAGAAGGATGAG	CTGTGATACATATGGCGGTCAATC
*Ptgs2*/COX-2	TGACCCCCAAGGCTCAAATAT	TGAACCCAGGTCCTCGCTTA
*Nos2*/iNOS	AGGTACTCAGCGTGCTCCAC	GCACCGAAGATATCTTCATG

## Data Availability

All data are available in the manuscript.

## Sample Availability

Samples of the compounds are not available from the authors.
